# Successful reduction in contaimination and an increase in blood culture collection rates, following introduction of adult blook culture packs

**DOI:** 10.1186/1753-6561-5-S6-P60

**Published:** 2011-06-29

**Authors:** R Bonnici, DM Xuereb

**Affiliations:** 1 Infection Control Unit, Mater Dei Hospital, Msida, Malta

## Introduction / objectives

Our hospital’s blood culture contamination rate within adult wards reached 5.2% prior to the introduction of adult blood culture packs. In addition, during 2009 the hospital’s blood culture collection rate was 31 blood culture bottles per 1000 patient days.

The aim of the study was to assess the impact of introducing a blood culture collection pack on the rate of blood culture collection and contamination.

## Methods

Standard in-house tailor made blood culture packs were devised and introduced across all 39 adult wards and units in hospital using a step wise approach in July 2010. The pack promoted the use of a closed blood culturing system using a vacutainer, and skin preparation using 2% chlorhexidine in 70% alcohol. The pack also included step by step photo instructions on how to take a blood culture from an adult using this new system. All junior doctors were given hands on training on how to use the pack and on basic principles of blood culturing.

Data obtained from laboratory information system were analysed to determine the contamination rates from 1^st^ January 2010 till 31^st^ December 2010. Blood culture contamination and collection rates before and after introduction of the packs were compared.

## Results

Over a 6 month period, the blood culture contamination rate was reduced from an average of 5.2% (235 of 4451 blood culture bottles) to 4.6% (280 of 6073 blood culture bottles) (*p*=0.038). The rate of blood culture collection was improved from 37.6 to 50.4 blood culture bottles per 1000 occupied bed days.

## Conclusion

The introduction of blood culture packs and appropriate training on blood culture collection are effective measures that increase blood culture collection and reduce contamination rates.

## Disclosure of interest

None declared.

